# Rethinking professional identity formation amidst protests and social upheaval: a journey in Africa

**DOI:** 10.1007/s10459-022-10164-0

**Published:** 2022-10-27

**Authors:** Mantoa Mokhachane, Ann George, Tasha Wyatt, Ayelet Kuper, Lionel Green-Thompson

**Affiliations:** 1 University of Witwatersrand, Johannesburg, South Africa; 2 Uniformed University of the Health Sciences, Bethesda, USA; 3 University of Toronto, Toronto, Canada; 4 University of Cape Town, Cape Town, South Africa

**Keywords:** African metaphor, Calabash, #Fees-Must-Fall, Professional identity formation, Social upheaval, Students’ protests, South Africa, Ubuntu

## Abstract

The under-representation of minoritized or previously oppressed groups in research challenges the current universal understanding of professional identity formation (PIF). To date, there has been no recognition of an African influence on PIF, which is crucial for understanding this phenomenon in places like South Africa, a society in which the inequity of the apartheid era still prevails. In addition, there is little data examining how social upheaval could impact PIF. This study uses interviews with medical students to explore PIF within the context of social upheaval during the 2015–2016 protests that rocked South Africa when students challenged asymmetries of power and privilege that persisted long after the country’s democratic transition. The combination of the primary author’s autoethnographic story, weaved into the South African sociohistorical context and ubuntu philosophy, contributes to this study of PIF in the South African context. The use of an African metaphor allowed the reorientation of PIF to reflect the influence of an ubuntu-based value system. Using the calabash as a metaphor, participants’ experiences were framed and organized in two ways: a calabash worldview and the campus calabash. The calabash worldview is a multidimensional mixture of values that include ubuntu, reflections of traditional childhoods, and the image of women as igneous rocks, which recognizes the power and influence on PIF of the women who raised the participants. Introducing an African ubuntu-based perspective into the PIF discourse may redirect the acknowledgement of context and local reality in developing professional identity.

## Introduction

The African experience is not reflected in the current constructions of professional identity formation (PIF). This reality and the ongoing under-representation of minoritized groups (in the South African context, the oppressed) in research present a challenge to the current universal understanding of PIF (Volpe et al., 2019; Wyatt et al., 2020, 2021a, 2021b). The formation through education and development of medical professional identity in Africa, and especially South Africa, may be rooted in the ubuntu philosophy (Letseka, 2012) (for more on ubuntu philosophy, see Box 1), as well as other critical theories (Balmer et al., 2021; Cornell & van Marle, 2015), all of which have characterized the liberation struggles in the society at large. Almost three decades after the transition to a democratic dispensation from the apartheid regime, the effects of apartheid, a colonial tool, continue in the ongoing experiences of inequity in South African society (Modiri, 2012). In seeking to understand the impact of social upheaval and protest action on emerging medical professionals, this study unearthed an uneasy relationship between a deeply divisive past and the tensions of a modern society. The exploration of students' experiences from the 2015–2016 period has forced deep reflection into the primary author’s experiences as a developing professional in the 1980s.

**Box 1:** Ubuntu philosophy

The foundation of ubuntu is perceived as motho ke motho ka batho (in Sesotho languages) and umntu ngumuntu ngabantu (in Nguni languages), which translates as a person is a person because of other people or a human being is a human being because of other human beings. Social interdependence is very important in an African community (Letseka, 2012). Mbigi (2005) in the Ubuntu Collective Finger theory gives an image of a hand, which illustrates that no matter how strong the thumb is, it is useless without the other fingers. He mentions five fundamental principles of ubuntu- respect, dignity, solidarity, compassion, and survival. Ubuntu epitomizes moral norms and values (Letseka, 2012). It is respecting others, their experiences and where they come from and the wisdom, they bring through that. Due to the collectiveness of ubuntu, gatherings in communities are valued as a crucial contribution to cultivating wellbeing	

Throughout this paper, the voice of the primary author (MM) will be foregrounded and is often expressed in the first person. MM is a Black, differently-abled (physical challenge) South African woman who studied medicine in the apartheid era, a time in which racial inequality, gender and disability discrimination intersected with multiple asymmetrical relationships of power and privilege to determine her life experience. This study, which analyses stories from senior medical students and recent graduates from a South African university, is juxtaposed alongside MM’s professional identity experiences thirty years ago. This paper has been written to allow for an authentic retelling of both the history of the primary author’s experience in developing a professional identity in South Africa and the contemporary lived experiences of medical students who participated in a national effort to bring equity to medical education. In areas where the writing pertains mainly to the first author’s story, the pronouns ‘I/me/my’ denote MM’s experiences; by contrast, the pronouns ‘we/us/our’ denote the voice of the full authorial team.

### A South African history of learning medicine

My journey to becoming a professional is distinctly different from what is reported in the prevailing PIF literature. Counter-storytelling is therefore important in breaking the mold of “dominant culture narratives” (Solórzano & Yosso, 2002) in medical education and training. I am a Black, differently-abled woman, a medical doctor working as an academic. An alumnus of an institution that, despite being the first to admit Black medical students in 1941, provided limited access to people of color through government quotas and ministerial consent (Digby, 2005). I attended medical school during the apartheid period in the 1980s and apartheid was overt during my training, but over time it has become covert, invisible, and difficult to confront. I will weave my narrative into the South African story and the history of education in my ‘beloved country.’ In doing so, I will argue that the South African context influences how PIF is acquired and developed and foreground the role of mental, emotional, social, and physical space in developing a professional identity. I do so partly because critical race theorists insist on “placing both the historical and contemporary issues in analyzing race and racism” in context (Solórzano & Yosso, 2002), hence the importance of telling the South African apartheid history in this paper.

As a colonial tool, apartheid was the formal legislative policy of the South African government between 1948 and 1990. The policy legislated the separation and governance of the society into racial and tribal groupings to entrench and preserve the privilege of a white minority. In the early 1900s, apartheid formalized segregation based on color, resulting in the racial classification of its people into white, Colored,^1^ Indian^2^ and Black (see Table 1). In the context of South Africa, race was socially constructed to create “superiority and dominance” (Solórzano & Yosso, 2002), using apartheid as an ideology embodying the inherent belief that whites are superior to other races (Lorde, 1992). The divisions among the different groups filtered into every sector of society, for example, schools, universities, hospitals, and means of transport, further promoting the racial divisions (Coovadia et al., 2009; Tobias, 1983). Given the unequal treatment of some groups, the United Nations declared apartheid a crime against humanity in 1966 (Coovadia et al., 2009; Dugard, 2008; Giliomee, 2003).

**Table 1:** Race classification

Racial classification in this paper				
	Indigenous	Mixed descent	Asian descent	European descent
South Africans	Black	Colored	Indian	White
Non-South Africans	Black African			

The control of education was a central tenet of the apartheid ideology, including limiting the progress of Black people in order to prepare them for lives of only servitude and manual labor (Giliomee, 2009), perpetuating white supremacy. Apartheid, in its nature, sought to minoritize each tribal group. In the tribal areas, this led to a series of ‘bush colleges’ for post-school education that developed over time. In 1951, a white institution, the University of Natal, created a Black section where Black medical students could be trained, but the mixing of Black and white people was not allowed (Noble, 2004). Entry of aspirant Black students into the established “white” universities was severely curtailed through the Extension of University Education Act of 1959. More than 20 years later, in 1976, a medical school for Black students was established (Haynes, 1995). The ongoing disparities under apartheid (Bickford-Smith, 1995; Saunders, 1992; Swanson, 1977), including educational inequalities, formed part of the motivation for student protest actions in the 1976 Soweto uprising,^3^ the 1980 school boycotts^4^ and, more recently, the 2015–2016 student movement called #FeesMustFall, which is central to the research presented in this paper. Residential townships and their associated schools were constructed using the apartheid racial classification (Coovadia et al., 2009). In the early 1980s, I received a ministerial permit to study at the local “white” university, the University of Witwatersrand, a place I was not meant to be. I chose that route for several reasons, one of which is that, with a strong awareness of where I came from, I felt empowered to turn the lives of my family around.

My identity was shaped first by the community that I was born into and second by the community I grew up in and the experiences I had along my journey to and in medical school. I developed poliomyelitis at two years of age. Poliomyelitis, a viral disease, left me with paralysis of the left leg. Growing up, I realized that occupying the education space was the only way to move my family out of destitution and change their position in the broader societal space. People around me were overprotective, often limiting my agency, due to what they perceived as the burden of my ‘disability’. Despite all their sorrows and sufferings, my family was sensitive about my disability, which led to a delay in my schooling. I was frustrated by not being allowed to attend school with others of my own age, but two of my older siblings became my teachers. They taught me how to read and write, and the dirt in our yard became our chalkboard.

Schooling for a differently-abled child comes with its challenges. I was mocked by other children. Walking with a foot in an equina varus position, with a tight Achilles tendon, was like being Achilles, the Greek warrior*,* ‘invulnerable in all of my body except for one heel’ (Chiodo & Wilson, 2006), having to walk barefoot in winter as I could not wear shoes. Later, I was admitted into the Black section (space) of a hospital to fix my left leg, to make it ‘shoe friendly.’ I was in a segregated hospital; white children had a beautiful playground while there was nothing on our side. The doctors, all white, would come to our ward for rounds, dressed in white safari suits and white coats. They were kind to us but could not communicate with us in our vernacular. I wondered if they did not see the injustice and the discrimination, which leaves me wondering now, more than 50 years later, about what influenced *their* professional identity formation.

Despite my experiences in hospitals, I was motivated to study medicine after the doctors had fixed my leg, perhaps even because of those doctors. My professional identity formation is richly imbued with empathy and respect through the intersections of my Blackness, my womanhood, and my disability in a country which had devalued all of these. Respect, a strong tenet of ubuntu, was a product of my upbringing. I was taught that you respect your elders by not addressing them by their first name, whether they are ten days older or ten years older than you. This principle from my childhood influenced my respect for seniority and hierarchy in medicine. This was often in conflict with my spirit of advocacy. I resolved this by challenging authority in a respectful way while developing a sense of ethical scholarship and advocacy during the early period of the human immunodeficiency virus (HIV) pandemic.

I was given a bursary for tuition when I entered the medical program in 1982. I travelled the 50 km from Daveyton (where I was allowed to live as a Black person) to the medical school, which was in a white area. Together with another Black South African young woman, we were given a room on the rooftop of a students’ block of apartments nearer to the school, masquerading as janitors in case the police came looking for Black people living there illegally. I felt unwelcome, not necessarily by the university at that time, but by the political system throughout South Africa.

We were eventually moved to a residence hall for Black students in Soweto (the sprawling black township on the outskirts of Johannesburg, about 30 km from the university). Glyn Thomas (a former university registrar and vice principal) was instrumental in setting up this residence for Black students and as such Glyn Thomas House (GTH) was constructed in his honor and, because of his support, Black medical students now perceive this house as an act of defiance (Wits Alumni Magazine, 2020). Living at GTH while studying to be a physician was transformative in every way possible; it was a social and a political hub that allowed us, as medical students, to grow exponentially into becoming community-responsive physicians. It was a place and a space where the next protest against apartheid would be discussed and planned. It was also a place that the system—and the special branch police forces used to enforce its power—watched closely, because anti-apartheid activists frequently met at the GTH (Wits Alumni Magazine, 2020).

During my training as a medical student, particularly during the internal medicine clinical rotations, as African students, we would share a meal together before every long shift (on call). We used to take turns preparing those meals for a shift, which fostered a sense of community that fueled our hard work and compassion during those long and extremely busy shifts. This reminded me of my home, where, as the five youngest children in the family, we would eat from the same large bowl not individual plates, stressing the importance of oneness; where celebrations would be held with music made from African instruments and the calabash (see more about the calabash in the next paragraph) being passed around to feed and quench the thirst of guests and family. During these gatherings, elders would tell us stories of where they come from and how they grew up. They would tell us how processes such as letsema (see Box 2) and lebollo (See Box 3) contributed to their growth and the growth of their communities.

**Box 2:** Letsema

Letsema means working together voluntarily for the common good. This is used in farming, building houses, etc., where the community is invited to assist one of the community members (Quan-Baffour and Lebeloane 2008). In this communal spirit, others sacrifice their time and sometimes their belongings to assist, for example, in cultivating someone’s land in preparation for ploughing. At the end of this activity, a meal or drink is offered to those who sacrificed their time. It is during these gatherings where a calabash, used as a feeding utensil, is shown as an embodiment of ubuntu

**Box 3:** Lebollo

Lebollo is regarded as one of the most important traditions in preparing youth for adulthood, namely, initiation. Lebollo is a ritual that marks the transition from girl to womanhood or boy to manhood. The initiation rites may vary from community to community. In South Africa, the initiation schools are situated away from villages and are underpinned by the values, beliefs, and practices of a particular community. Lebollo allows one to belong to a certain community of those that have gone through a rite of passage. Lebollo is expected to achieve cognitive engagement, virtue, confidentiality, appreciation of knowledge, respect and more	

A calabash,^5^ mentioned above, is a ubiquitous household gourd, used across Africa as a cultural expression, including at social celebrations of birth and other initiation rituals. The calabash transforms from a vegetable with a soft center into a tool with a hardened exterior, facilitating its utility in domestic life. This transformation is reminiscent of professional identity development and formation. Symbolizing transition, the calabash is an essential part of African communion, holding water on some occasions and traditional beer on others (Ellece, 2010). Women are responsible for making the calabash or African pot, giving it a gendered character. During ceremonies, the calabash is passed around for everyone to drink from the same source, creating a sense of oneness within the community. The calabash offers South African communities an image of holding the thoughts and pride of a community together in one place and the spirit of the calabash embodies the feminine attributes of nurturing and strength (Ellece, 2010). The calabash is an embodiment of ubuntu.

Observing the calabash being passed around during celebrations taught me the importance of oneness and planting a seed for growth in and through others. The image of the calabash and other African metaphors has been a matrix of the formation of my professional identity, which is based on oneness, respect, and selflessness. As a small community of African students, we made sure that our friends or clinical partners were not left behind. If one of us was not able to study for whatever reason, we would all go to the hospital to examine patients, present to each other and learn together.

My journey serves as the backdrop for the study we share in this paper. This study recounts a dynamic journey of self-identity development long before venturing into the professional space and the subsequent formation of professional identity in post-apartheid South Africa. The study centers around the Fees Must Fall protests of 2015–2016, a period of national student social unrest. The social disruption during the #FeesMustFall 2015–2016 movement underscored the tensions around exclusion on financial grounds from institutions of higher learning, experienced by individuals of color or lower-income class. During Fees Must Fall, which was part of a larger decolonial process (Ndlovu-Gatsheni, 2018), social media served as a vehicle of communication and mobilization during this major social disruption throughout South African higher institutions of learning. The major aim of the #FeesMustFall movement was to make quality education affordable and accessible to all, particularly poor Black students (Habib, 2019). The new South African government had not lived up to its promise to make education accessible to everyone (Cini, 2019a, 2019b; Habib, 2019; Langa et al., 2017; Mpofu, 2017). #FeesMustFall became the rallying hashtag for free higher education. The protests remain a watershed in South African higher education. There is little discussion in the literature about the formation of a professional identity in the context of social upheaval.

### Professional identity formation introduction

Holden posits that PIF is an integrative developmental process by which one establishes core values, moral principles and self-awareness, which all merge into individual values, beliefs and obligations (Holden et al., 2012). PIF is the confluence of identity development, professionalism and formation (Holden et al., 2012), a multifarious phenomenon influenced by clinical, non-clinical experiences, assumptions and ambient factors (Sarraf-Yazdi et al., 2021). Your personhood (personality), social structure and interactions play an important role in who you become or are becoming (Goldie, 2012). This is not an instantaneous but rather a gradual dynamic process. Based on the ring theory of personhood (RToP) (Radha Krishna & Alsuwaigh, 2015), becoming and identifying with the profession is shaped by various factors: the innate self, the individual, and relational and societal factors (Sarraf-Yazdi et al., 2021). This RToP, in a convoluted manner, echoes the saying in ubuntu philosophy, “I am because you are”, emphasizing that you are because of where you come from, what community you were brought up in (Letseka, 2012). In a community of practice (Lave & Wenger, 1991), individual, relational and collective identities shape one’s professional identity (Chandran et al., 2019). Each person brings their sociohistorical background into the PIF process and therefore this process is never the same for all trainees. Ultimately, professional identity is a confluence of professional and personal selves (Moss et al., 2014), which are also evolving. As Schrewe poignantly points out, the “I” of identity is irrevocably intertwined with and shaped by “the possibilities of being” that emerge from “one’s own historical moment” (Schrewe & Martimianakis, 2022, pg. 11).

Students enter professional training with a certain identity, shaped by their developmental communities, and are socialized into another identity as graduate professionals (Cruess et al., 2015). An individual’s professional identity formation is influenced by “who they are” at the beginning of their journeys and “who they wish to become” at their graduation (Cruess et al., 2015). PIF is defined as an idea of self, constructed based on attributes, beliefs, values, motives, and experiences (Slay & Smith, 2011). However, the idea of a single, universal notion of PIF in which students are forced to adopt the rigidly defined authoritarian and hierarchical identities of traditional medical teachers is being questioned (Helmich et al., 2017; Kaiser, 2002). The inflexible identity restricts the richness, diversity and uniqueness attainable in one’s identity as a medical student (Kaiser, 2002). This may ultimately harm *“*one’s confidence and sense of self-worth*”* as a doctor (Kaiser, 2002).

There are pitfalls in the PIF discourse which seem to result from notions originating in Western contexts of what a professional identity is and how it is created. The calabash represents something that is different from the Western perspective. It is a metaphor and a space to think about what ingredients are needed to create something that everybody can participate in and partake in. On the other hand, the Western ways of thinking about PIF are rigid or militant. They focus on the individual, not the context and not on the interrelationships. From the ubuntu perspective, these theories do not fit and PIF needs something that thinks about the relationship among these different things.

By expanding studies to include non-Western settings, the field may begin to unearth the nuances of how identities unfold rather than forcing them to conform to Western understanding (Helmich et al., 2017). It is important to explore PIF in different geographical and political contexts because PIF is a dynamic process (Cruess et al., 2015) that changes over time, molded by the contexts and spaces in which the professional development occurs (Cruess et al., 2019). Helmich proposes three approaches or considerations to answering the question “*Who am I.”* These considerations relate to your personal identity; your identity focusing on understanding yourself in relation to others, and your identity relating to a community (Helmich et al., 2017). In particular, Wyatt and colleagues argue that minoritized groups are often excluded from contributing to the dominant perspectives within the PIF canon (Wyatt et al., 2021a, 2021b) and challenged the dominant perspective that neglected to consider the intersections that characterize belonging to multiple minoritized groups based on gender, race and disability. Others, such as Conway-Hicks, interviewed participants who identified with “not from an advantaged background” and uncovered themes relating to “being and becoming a doctor” that displayed a hidden curriculum contribution, which silenced markers of socioeconomic *“*under-privileged” groups (Conway-Hicks & de Groot, 2019).

Previous studies explored “layered identities” through social relations such as “structured power, history*”* and others (Collins, 2015; Johnson, 2021), in which factors like race, gender and ethnicity lead to transient professional identities, in which individuals may be “privileged in one circumstance” while being marginalized in another (Crampton & Afzali, 2021). For example, a Black man may be privileged in the training space where he works with other Black patients and simultaneously be marginalized in a room full of white physicians. Considering “layered identities” within medical students’ professional identity is important because such aspects as race, gender, and ethnicity constitute forms of oppression in South African apartheid (apartness) and its ongoing legacy (Johnson, 2021). There is a paucity of data coming from Africa and South Africa on PIF, a continent that has been ravaged by colonialism, oppression, and war, and will undoubtedly add a different view of PIF.

This study describes and analyses the worldviews and experiences of medical students and recent graduates who trained during the #FeesMustFall 2015–16 protests, focusing on the time before and during their pre-clinical years in medical school. In so doing, we explore aspects of participants’ identities before joining higher education and their development of professional identities, considering both my experiences and theirs within the larger South African socio-political context.

## Methods

### Background and researcher reflection

I used my historical experiences as a lens for analyzing the data, and in doing so the participants and I co-created the initial interpretations, followed by an additional interpretation by members of the research team (LG, AG, and AK).

Before the student and recent graduates’ interviews, the first author reflected on and interpreted the meaning of her own lived experiences by first writing the story of her life and how she came to medical school (Laverty, 2003; Matua & Van Der Wal, 2015; Van Manen, 2016; Wojnar & Swanson, 2007). The entire team (LGT, AG, AK and TW) used the first author’s lived experiences as an analytical lens and analysed this personal story using critical theory (Solórzano & Yosso, 2002).

LGT and AG are both people of color who were students at the same institution as the first author during the ‘80 s decade. LGT and the first author were classmates and friends in medical school and shared some of the same clerkship rotations during medical training. All three of us experienced the tumultuous socio-political context of South Africa. To this study, LGT brought guidance, medical education, and social accountability expertise, while AG, with a PhD in education, brought structure to the supervision, and suggestions for theoretical frameworks and interpretive lenses, as well as emotional, educational, and technological support. AK and TW, both of whom had worked in North America, contributed a global perspective to the study. AK provided Global North expertise in the supervision, as well as guidance on theories and ways to structure the paper. TW’s role as a PIF expert was to raise questions about the context and data and to reflect on aspects of the first author’s story to create a coherent narrative (Naidu & Kumagai, 2016).

### Context and setting

This study was conducted at the University of the Witwatersrand, South Africa. It is a qualitative study with an African ontological grounding exploring medical students’ lived experiences during medical training, which took place between 2015 and 2020.

### Design

We used a qualitative, interpretive phenomenological enquiry (Shaw & Anderson, 2018, 2021), rather than a descriptive phenomenological approach (Reiners, 2012) because we were interested in understanding the meaning of students’ encounters. Further, I did not want to bracket (LeVasseur, 2003) myself during this process, as required by descriptive phenomenology. Additionally, in interpreting the medical students’ and recent graduates’ experiences of professional identity formation, we recognized early in the study that my experiences resonated with the students’ while I trained under apartheid. Therefore, we opted to engage with a “situated meaning of a human in the world” within the context of medical training in South Africa (Laverty, 2003).

### Participants

A purposive sample was drawn from senior clinical students and recent graduates, followed by a snowballing technique. The participants had been students in their first to fourth (MBBCh 1–4) years of the medical program during the #FeesMustFall 2015–2016 protests. All the student participants were in their final year (2020) of undergraduate training (MBBCh 6), as they had been in their first year during the protests. Two of the undergraduate medical students were graduate entrants into the medical-degree training while others had entered directly from high school. The medical graduates were in the first two years of their postgraduate training (internship) at the time they were interviewed. Student leaders were initially approached because of their active involvement in the #FeesMustFall movement; their recommendations then began the process of snowball sampling. An email was also sent to the 2020 final-year class, outlining the purpose of the study, and inviting those interested to take part. Those who showed an interest in the study were sent study information and consent forms for the interview and for audio recordings via email. There was also a psychologist available in case the participants needed counselling.

### Data collection

Interviews were conducted with students and recent graduates. Senior student participants were provided with data to facilitate the interviews when required. In total, 13 semi-structured interviews were conducted in 2020, of which eight were students in their final year and five were recent graduates. The interview protocol asked students to reflect on their experiences during and since 2015–2016, when the #FeesMustFall protests were at their height. The interviews explored each participant’s journey prior to their entry into the medical program (pre-admission), their journey through the program (student experience) and, for some, their journey beyond their training (the graduates). We used an interview guide to allow us to probe the following areas: the journey towards becoming a doctor, experiences during the #FeesMustFall protests, experiences of professionalism, and how #FeesMustFall impacted their professional identity development. The participant set consisted of five women, of which one was white and four were Black South African, and eight men, four of whom identified as Black South African, with one Black African (not from South Africa) and three white men. The extracts from the participants’ comments are identified using BF for Black female, BM for Black male, WF for white female and WM for white male (see Table 2). Three interviews were conducted in person, while ten were mediated through electronic platforms due to the pandemic lockdown restrictions (Skype, Zoom, Microsoft Teams and WhatsApp video call). The University of Witwatersrand Human Research Ethics Committee approved the study.

**Table 2:** Summary of Participants

	Demography	Source of funding
*Postgraduates*		
PG1BM	Black South African male	Government bursary, fully funded
PG2WM	White South African male	Government bursary, fully funded
PGBM, graduate entry	Black South African male	Initially missing middle, later privately funded
PG4BM	Black South African male	Government bursary, fully funded
PG5BF, graduate entry	Black South African female	Partially funded
*Students at the time of study*		
S1BF	Black South African female	Government bursary, fully funded
S2GWM, (graduate entry)	White South African male	Family funded
S3GWF, (graduate entry)	White South African female	Self-funded
S4BM	Black African male	Government bursary, fully funded
S5BF	Black South African female	Government bursary, fully funded
S6WM	White South African male	Government bursary, fully funded
S7BM	Black South African male	Government bursary, fully funded
S8BF	Black South African female	Government bursary, fully funded

### Data analysis

The interviews were transcribed verbatim and analyzed in MAXQDA 2020. Using an inductive approach, each transcript was analyzed as an individual case for the essence of its meaning and how participants’ experiences described influenced their being and becoming (Matua & Van Der Wal, 2015; Reiners, 2012). Coding was conducted line by line or at the paragraph level (Azungah, 2018) to identify natural meaning units. Codes were grouped into categories and categories into themes. The transcripts were read and coded first by all team members to create a common codebook. Each case (interview) was then profiled (i.e., the essence of each case was discussed and described by the first author and was further discussed and confirmed by the co-authors) and then compared for similarities and uniqueness. An in-case analysis and cross-case analysis was applied and “thematic connections” were made across cases (Bazeley, 2009; Wojnar & Swanson, 2007).

We used ubuntu to interpret the themes and their connections. Ubuntu is a South African ontological perspective, a different worldview from the Western notion of being. It originates from the concept of being (Muxe Nkondo, 2007) and can be translated as *personhood* or *humanness* (Kwizera & Iputo, 2011). The translation is not *being* as Heidegger describes it (Larsen & Adu, 2021) but an African way of being. Thus, it is not the Cartesian way of “I think, therefore I am,” but rather a more inclusive understanding of being summed up as “because we/you are, I AM.” Therefore, it is not a theory, but an ontological perspective and way of life, and is an entirely different way of knowing, seeing, and doing the world than what has henceforth been included in the PIF literature.

I used an ubuntu philosophy as an analytical lens to examine PIF among South African medical students, and in doing so applied metaphors to describe the findings. We used three South African languages, Sesotho, IsiZulu and IsiXhosa. For example, we use a hand metaphor similar to the one described by Mbigi in the collective finger theory (Mbigi & Maree, 2005), “matsoho a hlatswana,” which translates to “a hand cannot wash itself but depends on the other hand,” to communicate interdependence and mutual growth.

## Findings

To communicate our findings, we used the metaphor of a calabash, which represents a powerful transformation of a vegetable with a soft center into a tool with a hardened exterior to represent the transformation of South African students from undergraduate training to a fully-fledged physician. The calabash is meant to serve as an image of professional identity formation. As mentioned previously, Ubuntu originates from the African ontology, by which individualism is respected while interdependence is viewed as superior (Kronenberg et al., 2015; Muxe Nkondo, 2007). Despite the racial differences among participants, the principles of ubuntu were shared across indigenous and non-indigenous cultures, as they are in society (Muxe Nkondo, 2007), and participants entered the institution of higher learning with ubuntu home-based values, as well as a history of segregation and differential treatment for different races. They joined the campus calabash with ubuntu-based identities, socialized into another identity by the heat of #FeesMustFall, cementing the values of “I am because you are.” Using the calabash as a metaphor, we organized students’ experiences in two ways: a **calabash worldview** and the **campus calabash**, by first exploring students’ childhoods, then moving to university life, and then ending with the disruption caused by #FeesMustFall protests. Findings are presented in a story format with quotes from the participants.

### A calabash worldview

The findings show that students involved in the protests discussed the power of their upbringing and the ways in which the ubuntu philosophy shaped their personal and professional identity. In these discussions, we found that students discussed their reflections on a traditional South African childhood, the women in their families serving as rocks who raised, fed, and guided them, *Lebollo* (Sesotho for initiation), the traditional rites of passage from adolescent to adult, from student to professional, and *Ugqirha* (isiXhosa for doctor), using their bodies as healing instruments.

#### The values of ubuntu

Participants described bringing a sociocultural richness into the higher education spaces. Ubuntu embraces serving others without expecting anything in return (Kwizera & Iputo, 2011). Throughout this study, participants described caring for others as part of who they are as human beings and the ways it manifests throughout their journey to becoming physicians. The African metaphor of the calabash allows for a reorientation of PIF to reflect an ubuntu-based value system, in which the four-fold collective values of the ubuntu framework (i.e., survival, compassion, solidarity and respect) (Molose et al., 2018; Nussbaum, 2003; Schreiber & Tomm-Bonde, 2015) can be explored. For example, to express ubuntu, participants frequently discussed orienting themselves to the needs of others confirming that matsoho a hlatswana: “*helping others around you and seeing the change you make (S8BF”); “standing together”* (S2WM)*; and “looking at the needs of those around you”* (S3GWF). They also discussed having a strong sense of appreciating and respecting their elders, hierarchy in the South African context, and caring for their fellow human beings:

*The struggle is that we are brought up from families that are ubuntu driven, that are value-based and that value-based system in a sense of an elderly…is always an elderly. And the young should always toe the line in relation to that power dynamic. That we didn’t learn at Wits. (PG4BM)*

For many, the new university experiences were an opportunity to transform themselves, and participants expressed wanting to take what they learned in their upbringing and begin to interact with others who represent the patient population they would be treating. The love for diversity and their desire to know about other cultures reflected an essential part of their being. Some participants described feeling liberated from interacting only with those who looked, acted, and thought like them. For example, a participant who came from a very conservative community initially wanted to commute from home to the university campus every day. He was intrigued by the diversity he encountered during a tour of the residence halls and subsequently changed his mind about living there.

*When I came to Wits and to residence, it was completely different, everybody was English, there were different races, we were white, Black, Indian, multiracial, or multi-colored people, and everybody brought a piece of them to the table. (S6WM)*

This participant learned about other cultures and recognized that the thought processes of his peers were completely different from his way of thinking. He became immersed in the diverse calabash of language, culture, politics, and sociocultural richness, and expressed his growth as someone becoming better than previous versions of himself, “*I mean, if you had any conversations with somebody then it was clearly different to what I thought at the time” (S6WM).* In other words, the students brought their childhood value system with them, and mixed it into the diverse value systems and personal experiences with others to create a rich and nutritive environment.

#### *Mosadi ke lejwe la moralla*^6^* (woman is an igneous rock)*

The participants described the women in their lives as their rock. Male participants spoke fondly of their mothers and female participants spoke about the strong role mothers played in their lives. Some of the female participants were raised by single mothers and had a sense of women being stronger than men. There was a strong presence of the powerful voices of strong women, women who raised them, women who fed them, women who guided them and women who showed them how to be.

*And I think for me having like that kind of female background where my mom is my primary caregiver and my aunts are teachers who give so much, not only to their own children, but to their students…it motivated me, and it made me see like the power of kind of being over men and it made me kind of like a feminist. (S8BF)*

In South Africa, women balance the calabash on their heads filled with water, without holding or supporting it and walk for miles without spilling a drop. It is a mark of their strength and balance. Women hold their families together and nourish them just as they carry a calabash carrying water or food for communal nourishment. There is a saying in IsiZulu^7^ that celebrates the power and strength of women, “*Wa thinta abafazi wa thinta imbokodo*,” translated “*you strike a woman, you strike a rock.*” This sentiment was heard throughout the stories of these participants in that women are the rocks in their lives.

#### Lebollo—rite of passage

Choosing a university at which to study medicine was a well-thought-out process for many students. They did not just want to study medicine but wanted an experience that transformed and supported them, akin to a traditional South African rite of passage.

*Initially I wasn’t sure where I wanted to go, didn’t have a particular university in mind. For me it was just whichever university I could get in I would go to. And then Wits accepted me along with one or two others, and then I had to make a choice in the end and then Wits was the choice that I made. And yeah, I think I had quite a change in perspective initially…coming to Wits, it was a lot different than I actually expected, but also different in a good way. I think it exposed me to a wide variety of people, thought processes, and everything. (S6WM)*

Participants experienced their university life as a transition from adolescence to adulthood in ways that reflected a traditional rite of passage. They viewed the experience of becoming a physician in a way that positioned the knowledge they received about life, growth, resilience and how to deal with challenging matters, as a form of transformation. Although they did not use the term, their expression of growing is reflected in the concept of Lebollo, which is commonly used by the Basotho people meaning to “*facilitate transition to adulthood*”. *Lebollo* in Sesotho is a ritual that marks the transition from girl to womanhood or boy to manhood. In South Africa, these initiation schools are situated away from villages and are underpinned by the values, beliefs and practices of a community, which students made a conceptual connection to as they trained to be physicians.

Lebollo allows you to belong to a certain community of those who have gone through a rite of passage. Lebollo is expected to achieve cognitive engagement, virtue, confidentiality, appreciation of knowledge, respect and more. In lebollo, one is isolated in the initiation process and yet still with others, just as it is in medical training. In some cultures, one cannot practice as a healer until you have gone through this rite of passage, which is similar to practicing as a Western medical doctor; you must go through medical-school training before you practice.

#### *Ugqirha*^8^*—my body as calabash*

*Ugqirha* is the word for a medical doctor in the IsiXhosa language. Becoming a doctor was important to participants as they wanted to feed and nourish others into good health. This deep-seated yearning to heal others through their bodies sparked the desire in them to become doctors. Some of them were attracted by the inherent power that doctors hold and the doctors’ standing in the community, as this participant explained.

*I know that General Practitioner [Name withheld] you know, sometimes people just go to him, even if they are not sick, the guys they go to him for advice or…so there was this thing that a doctor had in the community that anyone else didn’t have. (PG1BM)*

Selfless concern for others was the core reason some participants wanted to join the profession, as they were eager to *“help people*” (S7BM), “*to make a difference*” (PG1BM), to be like an “*uncle who helped others*” (PG1BM) or an *“aunt, a teacher who helped or uplifted members of the community*” (S8BF). Seeing members of the community coming to show gratitude to teachers, nurses, and doctors in the community ignited in these participants the yearning to be like those respected members of their communities.

*So, like, that was kind of like my inspiration, like seeing how many teachers I had in my family, and I was just like being a doctor in its way is also becoming a teacher, I guess…ja…so that was like my…I didn’t have like a doctor-doctor, but I just had other people in my life which was more how working in the public system in South Africa can help others. (S8BF)*

Some participants were guided by parents’ desire for their children to be respected members of society, someone who exceeded what the parent might have achieved. A parent encouraged her daughter to study medicine because she felt that as a nurse she was not as respected as much as doctors in the clinical space.

*And my mom is a nurse and she’d always be like, you know, we get treated this way, this way, this way, you’ll be more respected as a doctor, all that stuff. (PG5BF)*

Family members who were healthcare workers were a motivating factor for some of the participants. Having a family member who either had a chronic disease or needed regular hospital check-ups was another motivating factor. Their motivation was either ignited by the respect for the doctors who looked after their family members or by the disgust that arose in the participants because of the terrible state of the public-health system. The participants’ intention was to change the way the public-health system is run in South Africa, to look after the poor who could not afford private health care.

*I grew up in a kind of like underprivileged family and I always use like the public health care system, and I was just like okay, I’m going to help people rather than go into like finance or the sciences, right. So, for me it was just kind of like if I work in the public health care system, I can make more changes. (S8BF)*

One of the participants, who is white, was guided or pointed in the direction of studying medicine by a White Sangoma.^9^

*So, I didn’t think I’d do it in Grade 11 but a sangoma told me that I should do it, was the first time when they told me that I should do it. I applied and then for some reason they decided to let me have a chance, so that was that. (PG2WM)*

These extracts reflect the multiple levels of motivation expressed for studying medicine, from the status it gives, the sense for healing, the sense of generational progress and ultimately the hearing of a calling.

In combination, participants’ stories invoked an image of students thrown into a large calabash where they were mixed with people of different backgrounds; they met people who do not look or speak like them but brought a rich diversity of experience that contributed to the creation of something nutritive for the whole community. Some lived on campus, while others commuted from other places. However, eight to nine months into 2015, university life changed. As a result of some students facing financial exclusion, a fire erupted in the calabash and students shut down the university, demanding that university fees fall. This period in their development as physicians is framed in two ways, ***Campus Calabash*** and ***Fees Must Fall, a divine interruption***.

### University life

#### Campus calabash

Initially, the students were living and thriving on the nutrients provided by the calabash as they transformed themselves into physicians. The calabash held all the ingredients for growing, which ebbed and swirled around to ensure that all flavors are expressed. Stepping into this space molded the participants, adding another layer onto their identity, taking them onto the next step of their formation. Different spices of life in this calabash seasoned them into different beings who had to adjust in giving and receiving knowledge. Challenged on a deeper psychological and emotional level, participants were iteratively softened and hardened throughout their time in training, laying the foundation of resilience and empathy. Some came from rural or township backgrounds and others from suburban areas. Some carried strong South African Black historical backgrounds that rendered them materially poor but socially enriched.

*I was coming from a rural school, a quintile 1*^10^*school in Limpopo province. And coming into university that was predominantly of those who were advantaged, we had to do a lot of adjusting, so my university years were challenging in that sense. And other factors that included social, economic, and family-related struggles were there, but mostly academically, those were the challenges that were related to the previous disadvantage. (PG4BM)*

One group came from privileged backgrounds, in communities with modest living standards and surrounded by high-achieving expensive schools. In this calabash, these privileged participants realized that, before they were thrown into this beautifully decorated vessel, they were not aware of the less fortunate:

*I was a white student from a private school. By no means was I able to self-fund at a private school and self-fund at Wits, but at the same time in my life I’ve had incredible opportunities of education. (PG2WM)*

Despite the varying levels of privilege and disadvantage, the students collectively experienced struggles transitioning from school to university. The participants had different levels of proficiency (i.e., technological proficiency) and had to embark on steep learning curves as they engaged in higher learning. They had to rapidly adjust to the tertiary environment. Their struggles were academic, economic, social, and family-related: “*We had to learn and adapt quite quickly. But the environment was supportive and nurturing”* (PG4BM).

They came into a big city from different spaces, from small towns and rural environments, all adjusting to new experiences. Being South Africans with a history of segregation, they were used to living with people who looked like them, who shared a similar culture and religion. This environment was a culture shock in a “good way”.

*I think I had quite a change in perspective initially…coming to Wits, it was a lot different than I actually expected but also different in a good way. I think it exposed me to a wide variety of people, thought processes, and everything, so I could say it was a bit of a culture shock initially, but in a good way. I mean, you learn to know about other people’s struggles and what they face and what they sacrifice to actually get where I was at that time as well. (S6WM)*

It was not only the white students who experienced shock, but also the Black students, as expressed by this participant, “*I never went to school with any other person who is not Black, so for me it was a racial shock when I arrived in an environment that was predominantly white”* (PG4BM).

The participants found their voice and the freedom of various forms of expressing themselves. For these participants, being on campus was an enriching curriculum on issues of life. In that environment, they experienced what life could be and gave them an example of what they would encounter in the communities as future doctors. For the first time, they saw men having sleepovers with men, men dressing as women and men wearing their hair in locks. Initially it was a shocking experience, but this taught them the value of freedom of expression. Understanding this is crucial to their development and caring for their future patients as free beings. This forced them to reflect on who they are, what they thought men or women should look like, dress like and behave. This is the epitome of learning as transformation.

*We had guys who would braid their hair in bright colors, guys who would wear dresses, guys who would have sleepovers with other guys, you know, and they were explicit about their sexual orientation. It was foreign to most of the people coming from rural areas, but by the time you come out of Wits you realize that you know what, as much as I was not exposed to that, that’s how people should be allowed to express themselves. (PG4BM)*

During the preclinical years, their role models were academics who looked like them and race was an important factor, more so for the Black students. Students also shared some formative experiences with only a subset of their medical school class. For example, students who stayed in a particular men’s residence spoke very fondly of that residence. According to the participants who stayed there, it was nurturing, where senior students mentored junior ones. A white participant who stayed there found it to be a place that contributed to his growth as a South African. He learnt more about other cultures, the history of the country and different cuisines. He was grateful to have lived in this residence as a white South African, as the experience he got was the training he needed before going into the community as a doctor.

#### Fees Must Fall, a divine interruption

In 2015, the start of the Fees Must Fall protests, created a divine interruption as it forced everyone to reflect on who they are, what they bring and their privilege or lack thereof, in the campus calabash, which started swirling and spiraling lives around to the next level of growth. This was a similar transformative process to lebollo, a process that made boys men and girls women. In the lebollo process, there is “knowledge production and transfer” which is meant to ensure the sustainability of nations (Maharasoa & Maharaswa, 2004). In a similar vein, the protesters attained knowledge about themselves, the calabash community, and the country. No one should be left behind was, in a way, the motto of the protests. Lebollo is the summit of the acculturation process, which Fees Must Fall facilitated to mold their formation and identities.

The following section is written for a non-South African audience in which the historical context has thus been interwoven into the narrative to clarify the intersection of the students’ stories with the development of their professional identity.

The participants expressed #FeesMustFall protests as a response to the tuition fee increase for 2015 in institutions of South African higher education. The protests were a fight for the basic human right to education after the government had reneged on promises made when it took the reins in 1994, when it had promised free education for all. Twenty-one years post-independence, mainly Black students were facing financial exclusion. In fighting for social justice, the students shut down the university to bring about that change. Participants felt that protests were a means of communication because they were not being heard. Fees Must fall started at the University of Witwatersrand (Wits) in October 2015. The movement spread through social media to all institutions of higher learning in the country, becoming a movement within a larger movement.

*I believe that I became a better advocate, firstly for issues, that were fighting for societal issues, and ultimately for patients’ rights as well. So, it changed, and as I alluded before, it gave us a confidence, and this feeling in that…and I would say for a lack of a better word, the entitlement of space. (PG4BM)*

*Because that’s what we were promised, or rather what our parents were promised, and they told us that, no, you my child will go to school for free because that was promised to us as well. So, it gives you that sense of anger that why are we supposed to go through such difficulties 26 years after democracy? (S7BM)*

While there was agreement in general about joining the protest, participants expressed major tensions in the 2015 first-year medical class about taking part in the protests. These disagreements were along racial lines, with the white students wanting to continue with classes. At that time, the privileged students did not see the need to protest. Despite all these tensions, some of the participants mentioned how much they have grown since 2015. For some they were conscientized about the needs of others and realized that the protests were a necessary way to make these needs known.

*So that’s when the racial divide started, because on the group we’d fight and everyone…like we’d be organizing marches, we’d be organizing, okay, which march are we going to as like students, what are we doing, what are we doing? And the white students would just be like, oh, no, we actually don’t want any part of that; we don’t want…like we’re okay. So, like, it was kind of like they didn’t want to learn, they didn’t want to open their eyes. (S8BF)*

According to the participants, protestors initially held meetings at the Senate House, the seat of university governance. Participants described how students renamed this building Solomon Mahlangu House in the spirit of decolonization. Participants reckoned that renaming these spaces was a gift to themselves, a reward for years of struggle. Occupying that space held an important place in their minds, hence the changing of the name to Solomon Mahlangu.^11^ Renaming this space gave participants a sense of belonging. They felt that they owned this space alongside the previously advantaged white students. In turn, the white students were being conscientized that the Black students were claiming, and are part of, the space.

*So, we felt that at that moment in time decolonizing that kind of space and renaming it to Solomon Mahlangu House was more of a gift to ourselves, to say, you know what, at least, inasmuch, yes, this institution may be against us, but we feel some sense of belonging. (S7BM)*

The protests illuminated shades of grey in both Black and white participants, also between male and female students, a mixture of privilege and deprivation. A large proportion of Black students, rich in the knowledge of the country’s history and culture, but lacking financially, were now facing exclusion. On the other hand, white students who were financially secure were now experiencing whiteness as deprivation: during the protests, some white, although emotionally invested and immersed in the movement, still felt excluded from the movement. Having not lived through the personal experiences of those in need, they lacked something; they felt incomplete, and they were trying very hard to be in that space. There was a lot of guilt, guilt for being, guilt for having funding, guilt for being privileged, and guilt for the way women were being treated. Women were sidelined, made to feel like they did not belong in the leadership of the movement.

*So, no matter how much I got involved in the protest, the more and more I realized that I was just on the side-lines, that I wasn’t a student who was deeply affected by it, or as affected by the increment stuff as some of my colleagues. (PG2WM)*

Participants felt that they did not get support from academics, some of whom were willing to continue with classes excluding a large proportion of students. Some of the activists in the medical program were victimized and labelled by academic staff as troublemakers. They were seen as disruptive and depriving the “paying” (PG2WM) students of their education. The situation became racialized, as those who were attending classes were mainly white. Subsequently, the attending students were forced out of their classes, a move that was initially taken as a violation of their rights. This action was also labelled as unprofessional. Those participants who had been forced out of the classes mentioned that they later realized the protests were necessary. This dawned on them when they learnt about the looming financial exclusions of some of their classmates and stressed that change had to come.

*It brings forth whether when you stand up for something you believe in denounces your professionalism, just because you’re not going with the flow, … I think a lot of minds that don’t always just do what they’re told…have always like in history been people that have brought about change. (S3WF)*

When students had exhausted every avenue and there was no headway, they elected to march to the Union Buildings,^12^ which are the seat of South Africa’s government. This was the most non-racial march ever seen in the country since the women’s march to the Union Buildings in 1956 (Legoabe, 2006). Participants said the students were united irrespective of colour or gender; they were marching and singing together.

*There are the strong, incredible, standing together, fighting together, marching together, pleading together, that was so positive and brought people so close together. (S2GWM)*

The march was peaceful until the President refused to come out to address them in person. Someone set a fire outside the Union Buildings and pandemonium erupted.

The campus calabash, lit by the flames of #FeesMustFall, instilled unexpected determination and growth in the students. Participants described being in a “war zone” (PG1BM), where they were learning to “*become better advocates*” (S8BF) as part of their professional responsibility. #FeesMustFall also challenged the way many of the students were brought up, having to suppress some of the values instilled in them during their upbringing, to win the war. For example, this participant struggled with the South African value of respecting those in power, *“I’m not attacking him because I’m disrespectful but I’m attacking him because of the power space he occupies, because of the office he occupies”* (PG4BM).

Through this experience, the participants, especially those who had come from positions of racial and financial privilege, realized that everyone has a story that needs to be heard and respected. They became activists and realized the importance of cultivating this role as a future physician, whether it be in a protest or in the clinical space.

## Discussion

I have lived through the period known as the South African apartheid, which silenced voices from the margins, and I used this experience in my research to understand the professional identity of students involved in the #FeesMustFall protests in 2015–2016. The participants’ stories about their involvement and the ways it impacted them echoed my own journey as a trainee during a time of silencing. It made me think about the advocacy that Steve Biko portrayed as a medical student (Biko, 2017). The results reveal to me personally that, as things changed into the post-apartheid era, they ultimately remained the same.

In writing this paper, I recognize that this is not a typical genre seen or employed in medical education, as this representation of what counts as a research study is somewhat different to the prevailing literature. I articulated the findings in a different way because, as a researcher, when I looked at the data, I saw shadows of myself in a different time and a different age. Therefore, I have intentionally taken a different approach in writing to underscore other ways of doing things, seeing the world, and communicating it, other than what has been sanctioned as legitimate ways of disseminating research in our profession. What counts as ‘acceptable’ within our disciplines is normed around Western constructs, which marginalizes and silences other ways of being. In writing differently, I am intentionally engaged in what Ramugondo supports as “everyday doing [that] disrupts societal dynamics of dominance” (Ramugondo, 2015). In other words, what we do in our everyday lives in the personal, clinical, and research space can either disrupt or perpetuate dominant forms that seek to keep some in perpetual oppression. By adding my voice to those normally heard in the Global North and working to legitimize different ways of doing things, my goal is to push our community to consider how to disrupt power structures that seek to keep some groups out, just as the political parties did during the apartheid era, and as rising tuition fees threatened to do during #FeesMustFall.

The results of this study underscore the role that childhood values play in creating a professional identity, and larger ontological systems that inform physicians’ worldviews. The buds of students’ professional identities emerge long before students enter clinical training or institutions of higher learning, in contrast to the “anticipatory socialization” (Brown et al., 2020) typically discussed in the PIF literature. Rather, molding occurs in their homes and communities through the principles of ubuntu and their experiences with the South African healthcare system. It also highlights how larger sociohistorical contexts (Wyatt et al., 2020) and contemporary social upheaval contribute to the development of one’s professional identity (Wyatt & Zaidi, 2022).

While much of the PIF literature concentrates on what happens in the pre-clinical (Niemi, 1997) and clinical (Cruess et al., 2016) years, our results show that students come to medical school with many of the attributes that are needed to become caring physicians. The foundation of their professional identity was already present even before they undertook their undergraduate years, which is in contrast to much of the PIF literature (M. D. Holden et al., 2015). By only considering Western notions of PIF and the role of culture (Volpe et al., 2019), researchers and medical educators are missing other conceptions of what contributes to a professional identity and the role of one’s personal background, such as gender and ethnicity, the yearning to give back to their communities and the culture of exclusion (Wyatt, et al., 2021a, 2021b). Therefore, one implication arising from this study is that medical educators should get to know their students on a personal level, to nurture the values, beliefs, and practices that are already developed by their home communities. Rather than shaping medical students to be the ‘right kind of doctor’ (Frost & Regehr, 2013), perhaps medical educators could rethink how we socialize medical students into the medical profession and minimise aspects of the hidden curriculum that lead to dissonance, hopelessness and apathy (Doja et al., 2016; Hopkins et al., 2016; Silveira et al., 2019).

One of the most important findings in this study was that students fought for social justice, a characteristic of being a physician entrenched in the physicians’ charter (Blank, 2002) as ‘health’ advocacy (Frank & Danoff, 2007), yet it was packaged in a different form. While much of the literature in North America talks about being an advocate at the level of the patient, in this study students were advocates for each other and an equitable training system.

Additionally, this study shows that #FeesMustFall became an important part of the hidden curriculum as participants learned things they could not have acquired in a classroom (Muxe Nkondo, 2007). Participants’ political consciousness was planted and grew, including as the privileged witnessed the difficulties experienced by others during this period of social upheaval. Despite the legacy of apartheid, they fought for survival as a collective, Black, rich, and poor, and people from the top side of society understood issues experienced from the underside. The privileged stood in solidarity with those at risk of financial exclusion out of compassion for fellow human beings. Whereas apartheid prohibited different racial groups from working together, the social upheaval of #FeesMustFall brought them together. Participants still experienced tension when they had to suppress their values to win the battle and employing disrespectful tactics as a political tool; however, the values participants embodied in the calabash were embedded in the participants. These findings underscore the need to further examine acts of professional resistance in medical education and the effect it has on trainees’ socialization into the profession (Ellaway & Wyatt, 2021).

There is some PIF literature within the Global North that points to the notion that sociohistorical contexts are important to the development of PIF in minoritized or previously ignored groups (Wyatt et al., 2020, 2021a, 2021b). In the American context, slavery, discrimination, and structural racism manifests in the way that PIF is expressed and develops among African Americans (Wyatt et al., 2020, 2021a, 2021b). On the other hand, the South African context brings a somewhat different context of developing PIF. Analyzing PIF as an “outsider within*”* (Hill Collins, 1986), as the first author has always felt in this Western-dominated space, reveals something that has not been seen in the PIF literature other than in work in the American South (Wyatt et al., 2020). In South Africa, PIF is influenced by the values of ubuntu, a history of apartheid and political strife, where space is highly guarded.

This introduces a very different sociohistorical twist, where PIF is developing within a context where one is fighting for and taking up space in order to belong and create opportunities for future generations. This is not just relevant to the Global North but relevant to everyone. This work therefore adds to the PIF literature the notion that there is a larger context to PIF, showing that everyone brings their own personal and social/cultural contexts to the professional identity development process, and events such as social upheaval can redirect the professional identity formation of medical students. While there are more women and Black medical students in the medical programs, much of the Western PIF literature in medical education remains rooted in the old dominant ideologies. Through this paper, we are bringing a different ontological perspective, when all we have is one perspective from the dominant ideological system. Members of groups from ‘other’ contexts—contexts other than those in which the current ‘universal’ definition of PIF was derived—experience a disjuncture because Western values clash with their own values. This allows us to re-imagine PIF as defined by space, time, and context, while also illuminating the limits of current conceptions that have only been acknowledged through studying dominant groups in the Global North.

Additionally, this study demonstrates that the PIF journey can be long, complex, and take unexpected turns. It starts at home for a South African child. It can be found in social upheaval—even in these moments when medical students were engaged in social upheaval and viewed as unprofessional. For these participants, fighting for social justice became a critical aspect of the identity formation process required for a physician. Their curriculum and identity formation were located in everyday life, not in a classroom or a clinical space.

We hope that our study demonstrates that medical education research and training should encourage the exploration of ways of thinking about what should be considered in studying PIF other than those common in the Global North, and the importance of other ways of knowing, being, and doing than what has been previously considered. Medical education spaces have become global stages, and data and ideas about how best to train physicians need to be interpreted from a wide range of cultural vantage points. In this global world, the training of doctors can also occur in spaces with a culture significantly different from that in which they were raised and socialized. As such, how then do we best support all physicians’ professional identity formation without erasing the richness and value of the varied culturally bound identities our learners bring with them to their training?

As this study shows, voices from outside North America, especially Africa, are not seen nor heard. Potential obstacles may include Africans experiencing silencing and bias in the publishing process because the stories they tell from this part of the world do not confirm to what has been established and accepted by various authors. However, in telling our own stories, using our own culture and metaphors to reach others who are not from Western culture, to whom the Western notion of PIF literature is foreign, has the potential to enhance identity formation for burgeoning physicians around the world.

## Footnotes

1 Mixed race.

2 People with their origins in the indentured laborer’s recruited in the Asian subcontinent, as a substitute for forced labor as a form of slavery.

3 https://www.britannica.com/event/Soweto-uprising.

4 https://www.sahistory.org.za/article/growing-social-unrest-community-mobilisation-strikes-and-student-protests-western-cape.

5 https://www.nationalgeographic.com/science/article/planet-calabash.

6 Woman is an igneous rock in Sesotho- one of the eleven official languages in South Africa.

7 IsiZulu is one of the eleven official languages in South Africa.

8 Ugqirha is a medical doctor in IsiXhosa, one of the eleven official languages in South Africa.

9 A traditional healer in South Africa. It is usually Black Africans that are trained by a healer but in recent times we have seen white people that also go through training. This is a calling from the ancestors.

10 South African public schools are categorized into 5 groups for purposes of allocation of financial resources. Quintile 1–3 are non-fee-paying schools while 4–5 are fee-paying schools. https://www.education.gov.za/Portals/0/Documents/Legislation/Call%20for%20Comments/NATIONAL%20NORMS%20AND%20STANDARDS%20FOR%20SCHOOL%20FUNDING.pdf?ver=2008.

11 https://www.sahistory.org.za/people/solomon-kalushi-mahlangu

12 https://www.sahistory.org.za/place/union-buildings-pretoria.
